# Case Report: Bilateral adrenal hemorrhage in a patient with systemic lupus erythematosus and antiphospholipid syndrome

**DOI:** 10.3389/fimmu.2025.1632069

**Published:** 2025-08-12

**Authors:** Yuwei Wang, Di Jin, Jiangang Pang, Wenlong Ju, Weiduo Nie, Rui Gao, Sheng-Guang Li, Ming Li

**Affiliations:** ^1^ Department of Emergency Internal Medicine, Weifang People’s Hospital, Shandong, Weifang, China; ^2^ Department of Rheumatology, Weifang People’s Hospital, Shandong, Weifang, China; ^3^ Department of Radiology, Yidu Central Hospital of Weifang, Shandong, Weifang, China; ^4^ Department of Urology, Qingzhou Traditional Chinese Medicine Hospital, Shandong, Weifang, China; ^5^ School of Traditional Chinese Medicine, Beijing University of Chinese Medicine, Beijing, China; ^6^ School of Clinical Medicine, Shandong Second Medical University, Shandong, Weifang, China; ^7^ Department of Rheumatology and Immunology, Peking University International Hospital, Beijing, China

**Keywords:** systemic lupus erythematosus, antiphospholipid syndrome, bilateral adrenal hemorrhage, adrenal insufficiency, thrombosis

## Abstract

This case report describes a rare and life-threatening complication of bilateral adrenal hemorrhage (AH) in a 15-year-old female with overlapping systemic lupus erythematosus (SLE) and antiphospholipid syndrome (APS). The patient presented with prolonged abdominal pain, low-grade fever, and lower limb pain. Imaging revealed bilateral adrenal hemorrhages, while laboratory investigations confirmed triple-positive antiphospholipid antibodies (ACA-IgG, ACA-IgM, anti-β2 glycoprotein I) and SLE-related serological markers. Management involved dual-pathway therapy: immunosuppression (prednisone, hydroxychloroquine) for SLE and anticoagulation (low-molecular-weight heparin, warfarin) for APS. Despite initial improvement, adrenal insufficiency developed, requiring glucocorticoid replacement. Follow-up demonstrated stabilized clinical status and reduced adrenal lesions. This case underscores the diagnostic challenges of AH in SLE-APS overlap and emphasizes the necessity of combining immunosuppressive and anticoagulant therapies to address both autoimmune inflammation and thrombotic risks. Early recognition and multidisciplinary management are critical to prevent adrenal crisis and improve outcomes in such complex autoimmune-thrombotic pathologies.

## Introduction

Antiphospholipid syndrome (APS) is an autoimmune thrombophilia characterized by recurrent arterial or venous thrombosis and/or pregnancy morbidity in the presence of antiphospholipid antibodies (aPL), including anticardiolipin (aCL), lupus anticoagulant (LA), and anti-β2 glycoprotein I antibodies. Systemic lupus erythematosus (SLE), another systemic autoimmune disease, is marked by multisystem involvement, production of various autoantibodies, and a broad range of clinical manifestations such as fever, skin lesions, and serositis.

While APS and SLE frequently coexist, with APS occurring in up to 30-40% of SLE patients, the adrenal gland is rarely mentioned as a target organ. When adrenal involvement occurs, however, it can be catastrophic. Adrenal hemorrhage (AH) secondary to APS is an infrequently recognized entity and can present with acute abdominal pain, hypotension, and shock—features that often mimic more common conditions like appendicitis or infection (1), In rare cases, AH may also be the primary manifestation of a cortisol-secreting adenoma (2). The resulting primary adrenal insufficiency (Addisonian crisis) is a life-threatening emergency.

Understanding AH in APS, SLE, and other connective tissue diseases (CTDs) is critical. Literature suggests that APS-related AH stems from thrombotic events within the central adrenal vein, while in SLE or other CTDs, vasculitis, microthrombi, or direct immune-mediated damage to the adrenal glands may play important role (3). Patients with combined APS and SLE may present with an even more complex clinical scenario, requiring concurrent anticoagulation and immunosuppressive treatment. Although rare, recognizing and managing AH early in these patients is essential for improving outcomes. Recently, the updated 2023 ACR/EULAR classification criteria for APS formally included adrenal hemorrhage as a microvascular manifestation, further underscoring the clinical significance of this rare yet severe complication (4). Therefore, although uncommon, early recognition and timely management of AH in these patients are vital for improving clinical outcomes.

In this report, we present a young female patient with SLE who developed APS and bilateral adrenal hemorrhage, subsequently progressing to adrenal insufficiency. We discuss the diagnostic challenges and therapeutic considerations. To better contextualize this case, we also review published cases of AH in APS and SLE providing an integrative perspective on clinical presentation, diagnosis, and management.

## Case presentation

A 15-year-old female presented to our hospital with a 20-day history of upper abdominal and lower limb pain. Two months prior, she had sustained a slip-and-fall injury, and over the past month, she lost approximately 5 kg. Family history was unremarkable. She began experiencing left upper abdominal pain without nausea, vomiting, or diarrhea, and mild left lower limb pain exacerbated by movement 20 days prior to this hospitalization. Three days later, she developed a low-grade fever (37.5°C). Meanwhile, her abdominal pain worsened and became a persistent dull ache accompanied by nausea and vomiting of gastric contents.

On initial examination, the patient’s blood pressure was recorded at 110/60mmHg. Physical examination revealed purplish-red rashes on the left finger and toe tips. Routine blood tests, biochemistry, thyroid function, and BNP were normal. Infectious markers for hepatitis viruses, HIV, syphilis, and tuberculosis were negative. Inflammatory markers, however, were elevated: PCT 0.136 ng/ml (ref ≤ 0.046 ng/ml), IL-6–40 pg/ml (ref ≤ 7 pg/ml), ESR 63mm/H(ref ≤ 20mm/H), and CRP 12.2 mg/L (ref ≤ 5 mg/L). Coagulation profiles showed INR (1.19; ref: 0.8-1.2), APTT prolongation (44 s; ref:22–38 s) and elevated D-dimer (2.48 μg/ml; ref ≤2 μg/ml). Serum electrolyte levels are within normal limits: Potassium 3.7 mmol/L (ref: 3.5-5.5 mmol/L), Sodium 143 mmol/L (ref: 135–145 mmol/L), Chloride 103 mmol/L (ref: 98–107 mmol/L), Calcium 2.32 mmol/L (ref: 2.15-2.55 mmol/L).

**Figure 1:**
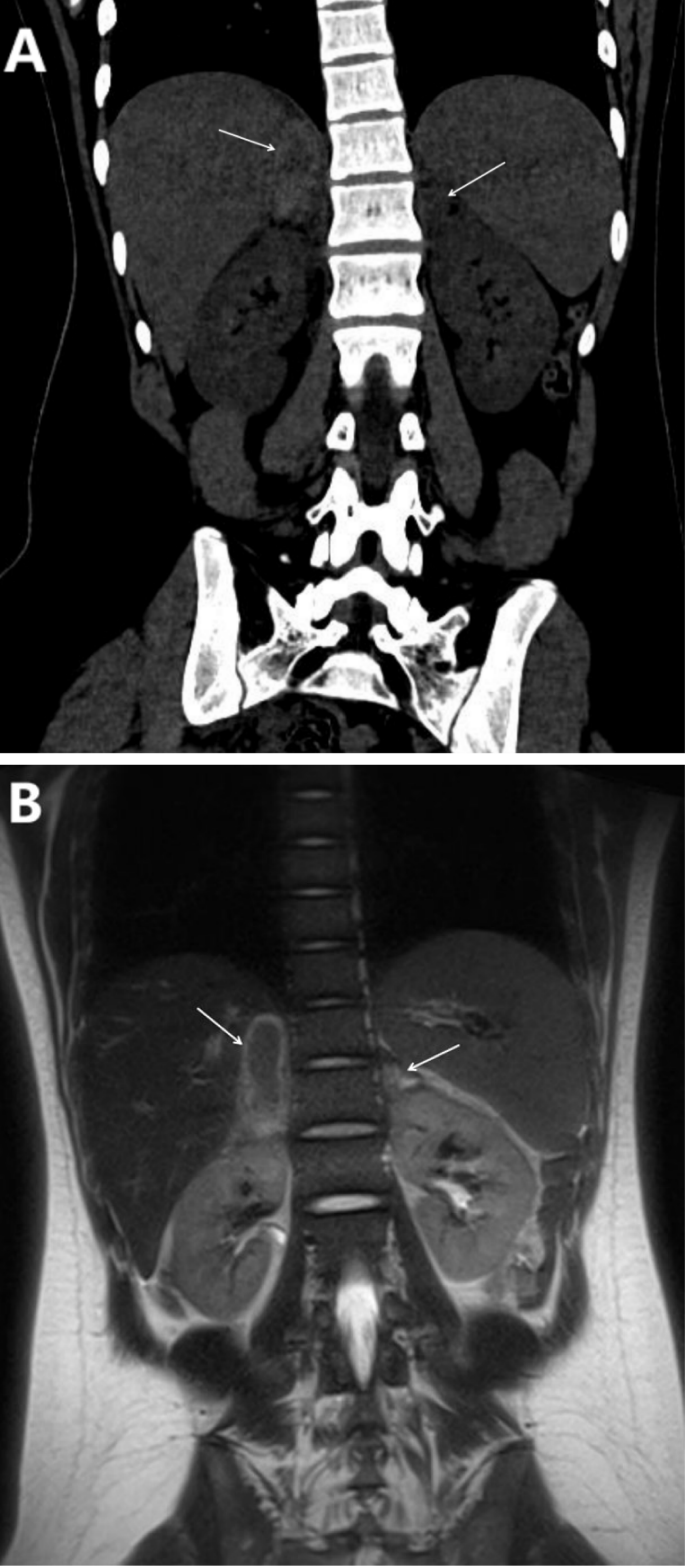
**(A, B)**. **(A)** The coronal view of the full abdominal CT scan (The adrenal lesion is indicated by the arrow). Right adrenal region: A patchy mass with mixed high and low densities, exhibiting increased peripheral density. It measures approximately 1.8 cm×4.7 cm, with a CT value of around 72 HU and well-defined boundaries. Left adrenal gland: A patchy isodense mass measuring approximately 1.6cm×1.0 cm, with a CT value of approximately 42 HU. **(B)** Adrenal T2-weighted MRI coronal view (The adrenal lesion is indicated by the arrow): An oval-shaped isointense to slightly hyperintense heterogeneous long T2WI signal lesion, surrounded by a hyperintense signal, is observed in the right adrenal region, measuring approximately 1.8 cm×4.5 cm with well-defined borders. A small focal lesion exhibiting a long T2WI signal is noted in the left adrenal region, measuring approximately 1.6 cm×1.0 cm.

Adrenal ultrasound demonstrated a 5.9×1.7 cm anechoic area in the right adrenal region and a 2.4 ×1.0cm in the left adrenal without contrast uptake. Lower limb venous ultrasound revealed thrombosis in the left superficial femoral and popliteal veins. Chest CT showed a subpleural high-density lesion in the left lower lobe and a small right-sided pleural effusion. Abdominal CT revealed bilateral adrenal hemorrhage (**Figure 1A**). Enhanced MRI of the adrenal glands confirmed bilateral adrenal hematomas with no significant enhancement, consistent with bilateral adrenal hemorrhage (**Figure 1B**).

Suspicion of bilateral adrenal hemorrhage and pulmonary infection emerged. Additional immunological testing found ANA high-titer positive (1:1000), while anti-Sm, anti-dsDNA, and anti-RNP antibodies were all negative. IgA, IgG, IgM, and complement levels were essentially normal. Triple positivity for antiphospholipid antibodies was confirmed: ACA-IgG 152.72 GPL/ml, ACA-IgM 70.89 MPL/ml, and anti-β2 glycoprotein 1 antibody 110.65 U/ml (all significantly above reference ranges). Lupus anticoagulant was also positive. These findings supported a diagnosis of APS (4). In addition, the presence of fever, chilblain-like rashes, pleural effusion, and positivity of antiphospholipid antibodies fulfilled the classification criteria for SLE (5).

The patient began receiving treatment included prednisone 40 mg/day and hydroxychloroquine 0.2 g/day one week after admission. For anticoagulation, she received low-molecular-weight heparin followed by warfarin with a target INR of 2.0-3.0. Following treatment, inflammatory markers returned to normal. A follow-up CT scan, conducted 20 days post-admission, showed a reduced right adrenal lesion and complete resolution of the left-sided abnormalities. The patient’s abdominal pain gradually eased, and she was discharged with prednisone 30 mg/day, hydroxychloroquine 0.2 g/day, and warfarin 1.5 mg/day. One month after discharge, however, she returned with dizziness, nausea, and vomiting after a cold exposure. Severe electrolyte disturbances ensued (K+ 6.1 mmol/L, Na+ 118 mmol/L, Cl- 86 mmol/L), refractory to initial corrections. Repeat imaging showed a right adrenal lesion. Endocrinology consultation diagnosed primary adrenocortical insufficiency (AI) secondary to bilateral AH. Hydrocortisone 100 mg twice daily intravenously was administered, later tapered to 100 mg once daily, and finally switched to oral hydrocortisone 80 mg/day. Long-term follow-up revealed stable remission on prednisone 2.5 mg every other day, no recurrent thromboses, and gradual tapering of glucocorticoids to minimal doses without flare-ups.

## Discussion

We report a young female with SLE who developed APS and bilateral adrenal hemorrhage. Initially presenting with abdominal pain and mild fever, she underwent extensive evaluation before AH was identified on imaging. Laboratory findings confirmed triple-positive APS and SLE, while imaging evolution and eventual adrenal insufficiency confirmed the diagnosis of bilateral adrenal involvement. APS is defined by vascular thromboses and aPL positivity (4). Adrenal involvement occurs due to thrombosis of the central adrenal vein, leading to infarction and subsequent hemorrhage. Clinically, patients may present with acute abdominal or flank pain, hypotension, nausea, vomiting, and electrolyte imbalances, notably hyponatremia and hyperkalemia, reflecting acute adrenal insufficiency (6). Recent evidence from Meade-Aguilar et al. further highlights the clinical complexity and severity of APS-associated adrenal hemorrhage (7). In their multicenter cohort and systematic literature review, they demonstrated that bilateral adrenal involvement and anticardiolipin IgG positivity are significantly correlated with primary adrenal insufficiency. These findings underscore the clinical importance of early recognition and aggressive management strategies, including both anticoagulation and hormonal replacement therapies, to improve patient outcomes. In isolation, APS patients experiencing AH often have straightforward thrombotic etiologies (8, 9). SLE, on the other hand, involves a broader immunopathology with vasculitis, inflammation, and potential complement activation^2^. While SLE commonly affects the kidneys, skin, joints, and serosal surfaces, adrenal involvement remains rare. AH in SLE without APS is less frequently reported (10, 11), and when it does occur, non-thrombotic immune mechanisms may be involved.

In patients with both APS and SLE (3, 6, 11–17), the clinical complexity is significantly heightened. The thrombotic tendency associated with APS interacts with SLE-driven inflammation, vasculitis, and potentially complement-mediated microangiopathy, creating a synergistic effect that may elevate the risk of adrenal infarction and hemorrhage. **Table 1** summarizes the clinical manifestations of 12 cases of APS and SLE combined with adrenal hemorrhage reported since 2003, including the present case. These patients often exhibit multi-system involvement, including skin lesions, pleural effusions, and autoantibody positivity, necessitating a dual therapeutic approach involving anticoagulation and immunosuppression.

**Table 1:** Clinical comparison of patients with systemic lupus erythematosus and antiphospholipid syndrome complicated by adrenal hemorrhage.

Patient (Ref.)	Sex	Age (yr)	Addision as first manifestation of APS?	Clinical manifestations of adrenal insufficiency	Adrenal CT/MRI appearance	Hyponatremia	Hyperkalemia	Cortisol(g/dL)	ACTH(pg/mL)	aCL	LA	Thrombocytopenia?	ANA	Anti-DNA Antibodies
Gao L et al. Case1 (3)	F	65	No	None	Bilateral AH	No	No	Not reported	Not reported	+	+	+	+	+
Gao L et al. Case2 (3)	M	45	No	Fatigue, Anorexia, Nausea,	Bilateral AH	Yes	No	Decrease	Elevated	+	+	Yes	+	+
Gao L et al. Case3 (3)	F	35	No	None	Unilateral AH	No	No	Not reported	Not reported	+	+	Yes	+	+
Gao L et al. Case4 (3)	M	34	No	None	Unilateral AH	No	No	Not reported	Not reported	+	+	Yes	+	+
Aldaajani, et al. (8)	M	43	Yes	Flank pain, dizziness, vomiting	bilateral adrenal glands bleeding	Yes	Yes	Decrease	Not reported	+	+	Yes	+	+
Zhang ZL et al. (11)	F	35	Not reported	Darkening of palmar creases, lips and buccal mucosa	Hemorrhage	No	No	Decrease	Elevated	+	+	Yes	+	+
Bouki K et al. (12)	F	30	Yes	Fever, lethargy, shock, hyperpigmentation, hyponatremia, hyperkalemia	Bilateral adrenal hemorrhage	Yes	+	Decrease	Not reported	+	+	Not reported	+	+
Diana et al. (13)	M	62	Yes	Adrenal crisis, abdominal pain, pigmentation	Bilateral adrenal hemorrhage (MRI: subacute phase)	Yes	+	Decrease	Elevated	+	+	No	+	+
Jiang et al. (14)	F	32	No	Abdominal pain, weakness, hyponatremia	Bilateral adrenal masses on CT (later cystic)	Yes	No	Decrease	Elevated	+	+	No	+	+
Ramon I et al. (15)	F	21	Yes	Acute	Enlarged, normal density	Yes	Yes	Decrease	Elevated	+	+	Yes	+	+
Riddell et al. (16)	M	42	Yes	Flank pain	Bilateral adrenal infarction (without hemorrhage)	Not reported	Not reported	Decrease	Not reported	+	+	Yes	+	–
Xu J et al. (17)	M	45	Yes	Fatigue, nausea, vomiting, low BP, hyponatremia	Bilateral adrenal hemorrhage, enlarged adrenal glands	Yes	No	Decrease	Elevated	+	+	Yes	+	+
Present Case	F	15	No	Prolonged abdominal pain, low-grade fever, and lower limb pain	Bilateral adrenal hemorrhage	Yes	Yes	Decrease	Elevated	+	+	Yes	+	+

Regardless of CTD background, AH typically presents with nonspecific symptoms (abdominal pain, nausea, vomiting, hypotension) and biochemical evidence of primary AI (hyponatremia, hyperkalemia). Imaging is crucial—CT and MRI can identify adrenal enlargement, hyperdense lesions, or hemorrhagic changes (6, 12–14). However, differences arise in the underlying mechanisms and optimal therapy. APS-related AH is primarily thrombotic; thus, anticoagulation is key (18). In SLE or other CTDs without APS, inflammation and immune-mediated damage may dominate, and immunosuppression is central (11). In patients with APS and SLE, both arms of therapy—anticoagulation and immunosuppression—are critical. This dual pathway reflects the multifactorial nature of adrenal pathology in these patients, who face vascular thrombosis in an environment of diffuse immune dysregulation.

Our patient’s course underscores the importance of tailored treatment. Identification of APS and SLE shifted the strategy: introducing anticoagulation stabilized the thrombotic risk, while prednisone and hydroxychloroquine controlled the underlying SLE inflammation. As adrenal insufficiency became apparent, hydrocortisone replacement was required to restore normal electrolyte balance and prevent crisis. Emerging data from case reviews show that patients maintained on appropriate anticoagulation and immunosuppression can have resolution or reduction of adrenal hematomas over time. However, long-term steroid replacement might be necessary if permanent adrenal damage has occurred (4, 12). Regular follow-up, repeat imaging, and hormone assays are recommended to monitor for recurrence or to guide gradual tapering of therapy.

One of the main challenges in diagnosing AH in APS or SLE is the nonspecific nature of symptoms. Abdominal pain, low-grade fever, and mild hypotension can mimic infections, appendicitis, or other acute abdominal conditions. A low index of suspicion can lead to delayed recognition, increasing the risk of adrenal crisis. Clinicians should consider AH in patients with APS or SLE who present with unexplained abdominal pain, hypotension, and electrolyte abnormalities. Prolonged APTT or positive aPL tests raise suspicion of APS-related vascular complications. Similarly, SLE patients with unexplained hypotension and electrolyte disturbances should prompt imaging studies to rule out adrenal involvement. The mortality associated with adrenal crisis is significant if untreated, but early diagnosis, aggressive supportive care, and targeted therapy can significantly reduce morbidity and mortality. Patients may eventually taper glucocorticoids if adrenal recovery occurs. Continuing anticoagulation for APS patients is essential to prevent recurrent thrombotic events, which could again jeopardize the adrenal glands or other organs.

## Conclusion

This case highlights a rare but critical complication of APS and SLE: bilateral adrenal hemorrhage leading to adrenal insufficiency. The clinical course demonstrates the importance of early imaging, comprehensive immunological evaluation, and prompt therapeutic intervention. There is a need for consensus guidelines regarding screening and management strategies for AH in patients with APS and SLE. Further research into the pathophysiology of adrenal involvement (particularly in APS and SLE) could identify biomarkers for early detection and guide targeted therapies to prevent adrenal damage before hemorrhage occurs. Early diagnosis and individualized management can prevent adrenal crisis and improve long-term outcomes.

## Data Availability

The original contributions presented in the study are included in the article/supplementary material. Further inquiries can be directed to the corresponding authors.
